# The first Boreal Sable (*Martes zibellina*, L., 1758) in the Pleistocene Arctic Siberia

**DOI:** 10.1371/journal.pone.0353165

**Published:** 2026-09-15

**Authors:** Gennadyy G. Boeskorov, Jiri Chlachula, Aisen I. Klimovskiy, Johannes van der Plicht, Albert V. Protopopov

**Affiliations:** 1 Diamond and Precious Metals Geology Institute, Siberian Branch, Russian Academy of Sciences, Yakutsk, Russian Federation; 2 Institute of Geoecology and Geoinformation, Adam Mickiewicz University, Poznan, Poland; 3 Environmental Research Centre, Stare Mesto, Czech Republic; 4 Mammoth Fauna Study Department, Academy of Sciences of the Republic of Sakha (Yakutia), Russian Federation; 5 Faculty of Science and Engineering, University of Groningen, Netherlands; Maria Curie-Sklodowska University: Uniwersytet Marii Curie-Sklodowskiej, POLAND

## Abstract

This study reports on the first Mustelidae find from the Arctic (NE Yakutia) sealed in intact Pleistocene cryolithic beds. A morphological analysis of semi-adult male sable remains—cranium and dentalia—shows a close affinity with the modern subspecies *Martes zibellina jenisseensis*. Absence of damage and attrition of the bone surface indicate *in-situ* burial of the ancient cadaver prior to soft-tissue decomposition. Just minor signs of microbial decay and post-mortem diagenesis changes in the osteological structure of the well-preserved skull and maxilla are consistent with long-term deep freezing. This fossil sable from the Ogorokha site (Central Indigirka, 68°23'N) is ^14^C-dated to > 45,000 BP, i.e., it is older than ~47,000 calendar years. The modern analogue of this typical woodland animal occupies today open taiga habitats in southern Siberia. The fossil sable shows that climatically moderate circumpolar interstadial ecosystems are conserved in local yedoma deposits. This unique find indicates a former geographic distribution of sable far north into the present sub-arctic and arctic territories, and supports the existence of biotically rich forest refugia during the Last Glacial.

## Introduction

**Fig 1:**
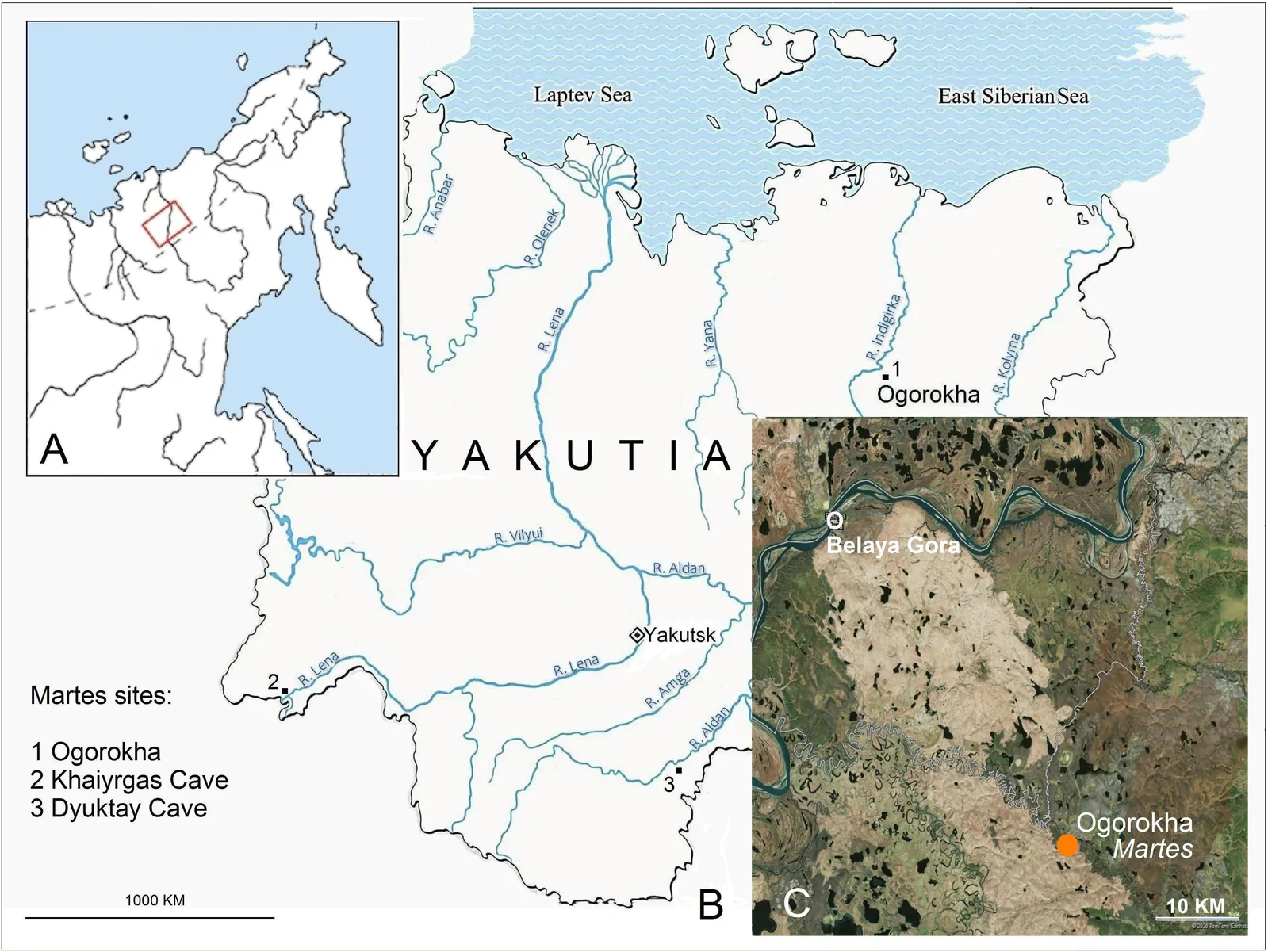
1A. Map of the study area; 1B. Geographical location of the Late Pleistocene sable sites: 1 the present find *(Martes zibellina)* – Ogorokha Site; the former records (*Martes* sp.) – 2 Khaiyrgas Cave, – 3 Dyuktay Cave (2–3) [1]; **1C.** Location of the Ogorokha River valley and the investigated site within the central Indigirka (satellite photo: Earth Explorer, USGS).

Northeastern Siberia (Fig 1A) still remains one of the least explored parts of the World because of its remoteness and the large (~5 million km^2^) geographic space. The area, covered by continuous permafrost [2,3], experienced major climate shifts in the past. Long-term variations in temperature and atmospheric humidity predetermined distribution of the Late Pleistocene syngenetic yedoma ice in the sub-Arctic and Arctic [4]. Present climate change has affected the integrity of cryolithic grounds that developed during the coldest (stadial) stages of the Last Ice Age [5]. Environmental changes caused geomorphic (gravity-flow and thermokarst) processes comparable with modern ones [6]. Thaw of massive syngenetic ice formations prompted major transformations in the regional and local landscape settings and natural habitats [7,8]. These changes, particularly active during the mid-last glacial interstadial (MIS 3; ~ 58,000–25,000 years ago) enabled an expansion of the southern biota (flora and fauna) into forest tundra and tundra due to raised mean annual temperatures [9].

Sable (*Martes zibellinа* L., 1758; Mustelidae, Carnivora, Mammalia) is a typical inhabitant of the boreal lowland and mountain coniferous forests. This species is currently distributed throughout Eurasia [10,11]. The study area is inhabited by a taxon interbred from several populations brought in to restore the former population present in the 1940–50's [12]. Records of Pleistocene sable are extremely rare. An ancient (Middle Pleistocene) ancestral form of sable (*Martes* sp.) is mentioned in the list of mammal species discovered in the Zhoukoudian-1 Cave in North China, and from the Late Pleistocene layers of the Xitaiping Cave near Beijing [13,14]. On the territory of central and eastern Russia, some skeletal fragments were found in the Urals and the Altai Mountains [15], in Western Siberia [16], and in Primory’e in the Russian Far East [17].

There is very little information about the Late Pleistocene remains of *M. zibellina* in Yakutia (East Siberia). A. K. Kasparov [18] and G. G. Boeskorov [19] identified several sable bones from the Upper Paleolithic layers (Units 5–6) in Khaiyrgas Cave. A sable bone from Unit 4 (8,085 ± 80 BP) indicates an early Holocene age [20]. The likely remains of *M. zibellina* [1] were found in the Dyuktayskaya Cave in the upper reaches of the Aldan River (Unit 7b) fixed by the ^14^C dates of 12,100 ± 120 BP and 12,690 ± 120 BP [21], and Geographical Society Cave from the MIS 3 deposits [17].

This article reports and discusses a well-preserved find of a complete cranium of *Martes zibellinа*, L., 1758 from the central Indigirka River area—the Ogorokha Site. It is the first, the oldest, and the northernmost located sable species discovered in Siberia. Morphological, chronological and environmental aspects of the sable specimen are discussed.

### Study area

The location of our investigations is the Ogorokha River valley, which is the right tributary of Indigirka in the Belaya Gora Administrative District—a polar region of Yakutia (Fig. 1B). The central part of the basin opens to the north into the Indigirka–Kolyma Lowlands bordered by the Momskyy Range in the South, and the Verkhoyansky and Cherskogo Mountains (800–3000 m asl.) in the West and East, respectively [22] (Fig. 1B). The valley cuts through a low- relief (100–300 m asl.) erosional topography. The regional Cenozoic geology is structured by pre-Quaternary fluvial, colluvial and lacustrine deposits [23,24]. The broadly distributed Late Pleistocene formations—defined by sandy-gravelly beds and silty-clay gravity facies—are known for numerous Pleistocene palaeontological data [25,26]. The rich fossil remains are primarily associated with the Last Ice Age cryogenic silty-clay yedoma and riverine alluvia [3,27].

The ground temperature in the broader Abyiskaya Lowland is between –3 and –10°С; the thickness of syngenetic (permafrost) zone is 200–500 m [28]. The active layer (seasonally degraded permafrost) reaches 0.5–1.2 m below the surface depending on the bedrock. The present arctic climate is characterized by extreme seasonality with low annual precipitation rates (150–200 mm) and high seasonal temperature differences. The average value is –31ºC in January, and +14ºC in July; the mean annual air temperature (MAAT) is –11ºC. Northern boreal forests consist of Larch *(Larix cajanderi)*, along with willow (*Salix* sp.) and alder *(Alnus* sp.) found in humid valley settings. In drier places it transgresses into open parklands. Sedge-meadows around boggy ponds and mosaic herb-meadows with dwarf birch *(Betula nana)* illustrate the north-retreating tundra-steppes due to the current MAAT rise in Northeastern Siberia [6].

## Materials and methods

The subject of investigations is an isolated find of a cranium of *Martes zibellina* L. (reg. Og-14–1) found in August 2014 in Pleistocene cryolithic deposits, the bulk of which consists of surface cover of the Ogorokha valley. This finding was reported in a short preliminary communication [29]. Detailed contextual geological and geomorphological investigations followed a regional stratigraphic correlation with other palaeontological and Palaeolithic archaeological sites [30]. A contextual sediment sampling and collection of organic materials (macro-botanical and faunal skeletal remains) were performed using standard procedures. The palaeogeographical and palaeoenvironmental studies of the Ogorokha locality were carried out at the Institute of Geoecology, Adam Mickiewicz University, Poznan, Poland.

The reported find of *Martes* was carefully removed from in situ position from the exposed cryogenic deposit subjected to progressing seasonal thaw. For transport, the specimen was packed and kept in a frozen state, for studying the sample at the Mammoth Fauna Research Department, the Academy of Sciences of the Republic Sakha, Yakutsk. The species determination is based on the analysis of the stored and published materials of the fossil/modern Siberian Mustelidae [1,15]. Taxonomy and taphonomy of the associated Pleistocene fauna mammal remains completed the reconstruction of the Last Ice Age fauna community.

Radiocarbon dating of the fossil bone was performed at the Center for Isotope Research, Groningen University (the Netherlands). ^14^C dating of the accompanying fossils from the same formation was provided by the Poznan Radiocarbon Laboratory (Poland) [30] yielding a consistent regional chronology. Here we report shortly on the isotopic analysis of the *Martes*.

The concentration of Radiocarbon (^14^C) and of the stable isotopes ^13^C and ^15^N are measured for collagen extracted from the bone. The reparation method follows a protocol originally developed by R. Longin [31]. To extract the collagen fraction from bone samples, fragments are first subject to a mild ABA (Acid-Base-Acid) pretreatment [32,33]. The extract is exposed to NaOH to eliminate humic acids, and subsequently transferred into gelatin. The prepared gelatin is combusted by a so-called Elemental Analyser (EA) into gas (CO_2_ for ^13^C and ^14^C analysis, and N_2_ for ^15^N analysis).

The stable isotope contents are reported in delta values, which for ^13^C is a measure of the difference of the ^13^C/^12^C isotope ratio of a sample and that of a standard (or reference) material:

δ^13^C = (^13^C/^12^C)_sample_ / (^13^C/^12^C)_reference_ – 1 (x1000‰)

For ^13^C, the reference is an international standard limestone known as Pee Dee Belemnite, or V-PDB which has an accurately measured absolute ^13^C concentration.

For ^15^N, this is:

δ^15^N = (^15^N/^14^N)_sample_ / (^15^N/^14^N)_reference_ – 1 (x1000‰)

and the reference is ambient air.

For absolute concentrations of the rare isotopes in the standard materials we refer to [34] and compiled references therein. The δ-values are expressed in permil (‰). The measurement precision is 0.1‰ and 0.2‰ (1) for δ^13^C and δ^15^N, respectively. The δ-values are measured by IRMS (Isotope Ratio Mass Spectrometry). The instrumentation consists of an EA (Elementar Vario Isotope Cube) and an (IsoPrime 100^TM^), coupled to the EA.

The ^14^C concentration of the collagen is measured by Accelerator Mass Spectrometry (AMS). For the *Martes* sample the instrument was a 2.5 MV Tandetron [35]. ^14^C dates are reported by convention in BP. The convention comprises the use of the half-life value of 5568 years, the standard reference activity (95% of the activity of the specific batch of Oxalic Acid nr.1 in 1950), and a correction for isotopic fractionation to δ^13^C = −25‰ [36]. The ^14^C dates in BP require calibration into calendar years, which are reported in cal BP, i.e., calendar years relative to 1950 AD. Calibration is done using the most recent calibration curve IntCal20 [37].

### Site geology, chronology and palaeontology

**Fig 2:**
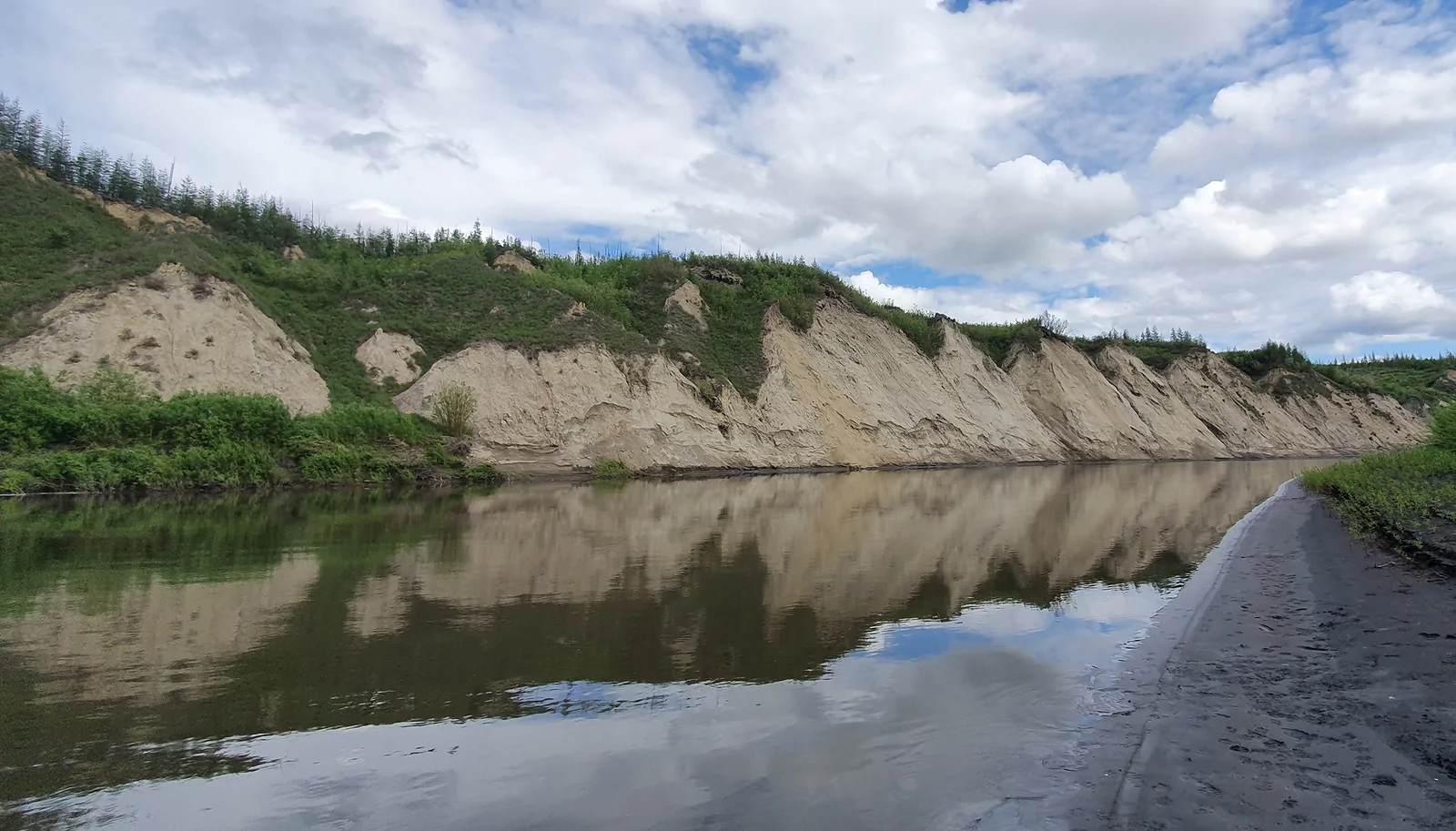
The Late Pleistocene fossiliferous cryolithic sections in the Ogorokha valley.

**Fig 3:**
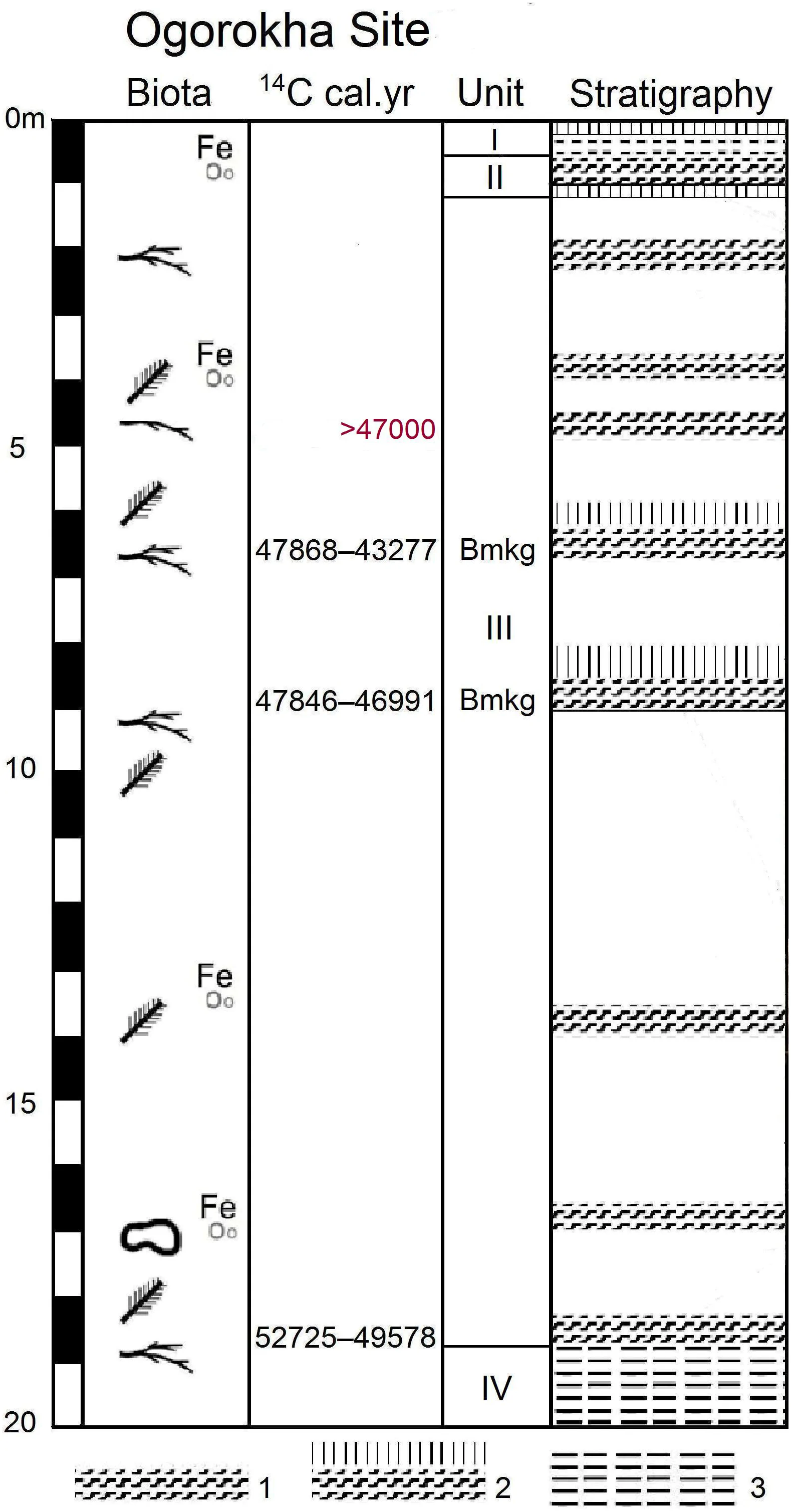
Ogorokha Site geology. Legend: Unit I – Present forest-tundra soil; Unit II – Sandy sub-surface pedogenic horizon, colluviated and cryogenically distorted; Unit III – Series of colluviated beds of sandy silts, interstratified by gleysolic horizons, incorporating fossil wood and organic detritus; two brunisolic palaeosols (Bmkg horizon); Unit IV – Humic, organic-rich overbank or backwater alluvium with fossil leaves and twigs. Legend: 1– ferruginous horizons, 2 – MIS 3 palaeosols, 3 – alluvial clays. The fossil *Martes zibellina* find is fixed by the ^14^C date >47,000 cal BP (GrA-62462); other radiocarbon dates [38].

The cranium of the Pleistocene sable was found in an excellent condition on the left bank of the Ogorokha River—a right tributary of the Badyarikha River within the middle reaches of Indigirka River (Fig 1C). The site of discovery (68°23’09” N, 146°82’22” E) is a natural erosion of the present riverbank exposing the Last Ice Age yedoma deposits (Fig 2). Here, the remains of mammals of the mammoth fauna are collected in large numbers released from stratified cryogenic beds, following seasonal permafrost thaw. The Quaternary sections (up to 10 m high) along the left river bank are formed by gray silty clays, alternating with layered sandy and sandy-silt deposits (Fig 3). Massive ancient syngenetic ice veins and lenses cut through the lower 2/3 of the formations. The reported fossil find was buried ~5 m below the present surface about 20 m from the active river bank. Recent chronostratigraphic and palaeontological studies securely fix the contextual cryogenic bodies as Late Pleistocene; the principal fossiliferous beds are ^14^C-assigned to the early MIS 3 interstadial interval [30].

The bone dates older than 45,000 BP (GrA-62462). This is the upper limit of the ^14^C dating method for bones [35]. Beyond this limit, the ^14^C radioactivity of samples can generally not be distinguished from the natural background activity. In terms of absolute age the calibration curve IntCal20 [37] shows that the bone is older than ca. 47,000 cal BP. The measurement corroborates the dating of other Pleistocene fauna remains uncovered from the same colluvial formation at the Ogorokha locality [30]. The bone collagen quality was excellent, as expected for permafrost conditions: the Carbon content is 43.8%, the Nitrogen content 15.8% and the atomic C/N ratio 3.2, all within accepted range. The measured stable isotope values are δ^13^C = −20.19‰, and δ^15^N = 8.63‰. These single-find values are not used for an interpretative basis. Forty thousand-year cryolithic burial in the yedoma deposits under very low and stable freezing temperatures allowed for the unique conservation of the fossils.

The abundant fossil records incorporated in the cryolithic yedoma document the most productive part of the MIS 3 interstadial (58,000–42,000 years ago) in terms of the flora and fauna diversity. This is also confirmed by the ^14^C date of the *Martes* sp. reported here. The well-preserved cranium was found together with typical representatives of the mammoth fauna mammals: Leporidae – *Lepus tanaiticus* (Gureev, 1964); Ochotonidae –*Ochotona hyperborea* (Link, 1795); Mustelidae – *Gulo gulo* (Linnaeus, 1758); Canidae – *Canis lupus* (Linnaeus, 1758), *Vulpes vulpes* (Linnaeus, 1758); Felidae – *Panthera spelaea* (Goldfuss, 1810); Ursidae – *Ursus arctos* (Linnaeus, 1758); Cervidae – *Rangifer tarandus* (Linnaeus, 1758), *Cervus elaphus cherskii* (Boeskorov, 2005); Bovidae – *Bison priscus* (Bojanus, 1827); Rhinocerotidae – *Coelodonta antiquitatis* (Blumenbach, 1799); Equidae – *Equus lenensis* (Rusanov, 1968); Elephantidae – *Mammuthus primigenius* (Blumenbach, 1799). The local fossil fauna biostratigraphy, taxonomy and taphonomy enable close Ice Age ecosystem interpretations [30]. The Ice Age sable has not been previously recorded within the Arctic.

### Morphological description

The specimen of *Martes zibellina* L., 1758 was uncovered *in situ* in frozen yedoma deposits in a very good state of preservation comparable to recent osteological remains of the animal. The bones of the skull are practically intact indicating a long-term (Ice Age) cryolithic burial.

**Fig 4:**
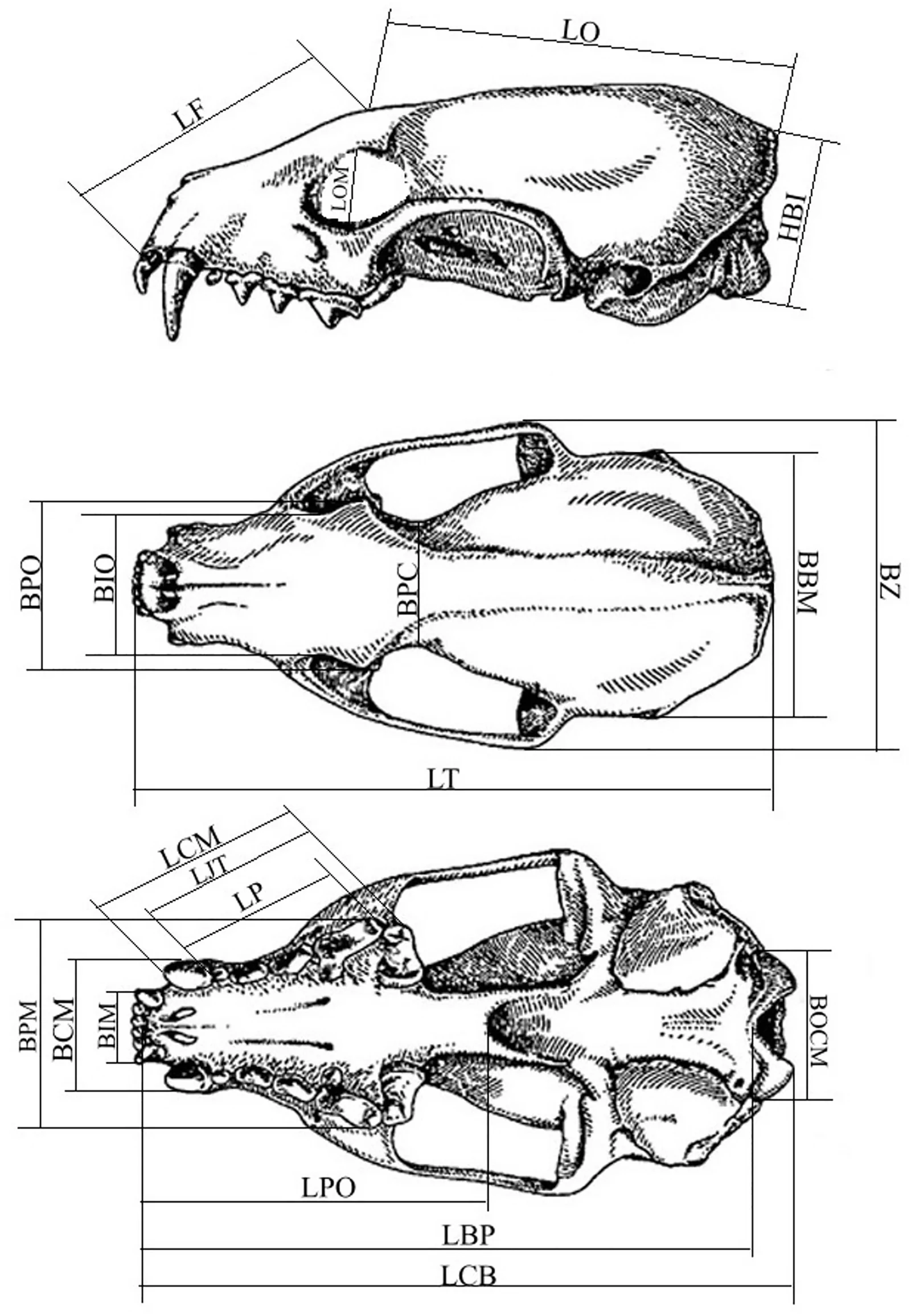
Sable skull morphology. A – lateral view. B – dorsal view. B – ventral view.

The following descriptive morphology abbreviations are used (Fig 4A–4C):

Teeth measurement abbreviations: LT – Total length (Akrokranion – Prosthion); LCB – Condylobasal length (aboral border of the occipital condyles – Prosthion); condylobasal length, LBP – basal length (Basion–Prosthion) / main length, LF – facial length (Frontal midpoint – Prosthion); length of the facial part, LO – occipital length (Upper neurocranium length: Akrokranion – Frontal midpoint); LPO – palatal length (Staphylion–Prosthion); LJT – length of tooth row P1–M1 (measured along the alveoli on the buccal side); length of tooth row from P1–M1; LP – length of tooth row from P1-P4; length of tooth row P1–P4; LCM – length of tooth row C1-M1; LOM – maximum length of the orbits (greatest inner length of the arch); maximum eye socket length, HBI – height from basion to inion; The measurements of the dental arch: L – mesiodistal length; B – buccolingual width; BMts – width at the level of the metastyle, LPa – length of the paracone.

Skull parameters: The two pointers of the slide gauge are placed basally on the basis of the skull (on the basioccipital) and dorsally on the highest elevation of the sagittal crest; HBI – height of the occiput from the lower edge of the occipital foramen; ВIО – interorbital breadth; width between the orbits; ВРО – breadth between the postorbital processes of the frontal; width of the supraorbital process; ВРС – breadth at the postorbital constriction; ВВМ – maximum breadth of the braincase; ВZ – breadth between zygomatic arches; VSM – maximum width in canines; BIM – maximum breadth of the incisor teeth; BPM – maximum breadth of the palate (measured across the outer borders of the alveoli P4); BOCM – maximum breadth at the occipital condyles. Measurements were performed according to K. Wagner [39] and A. von den Driesch [40].

#### Cranium.

**Fig 5:**
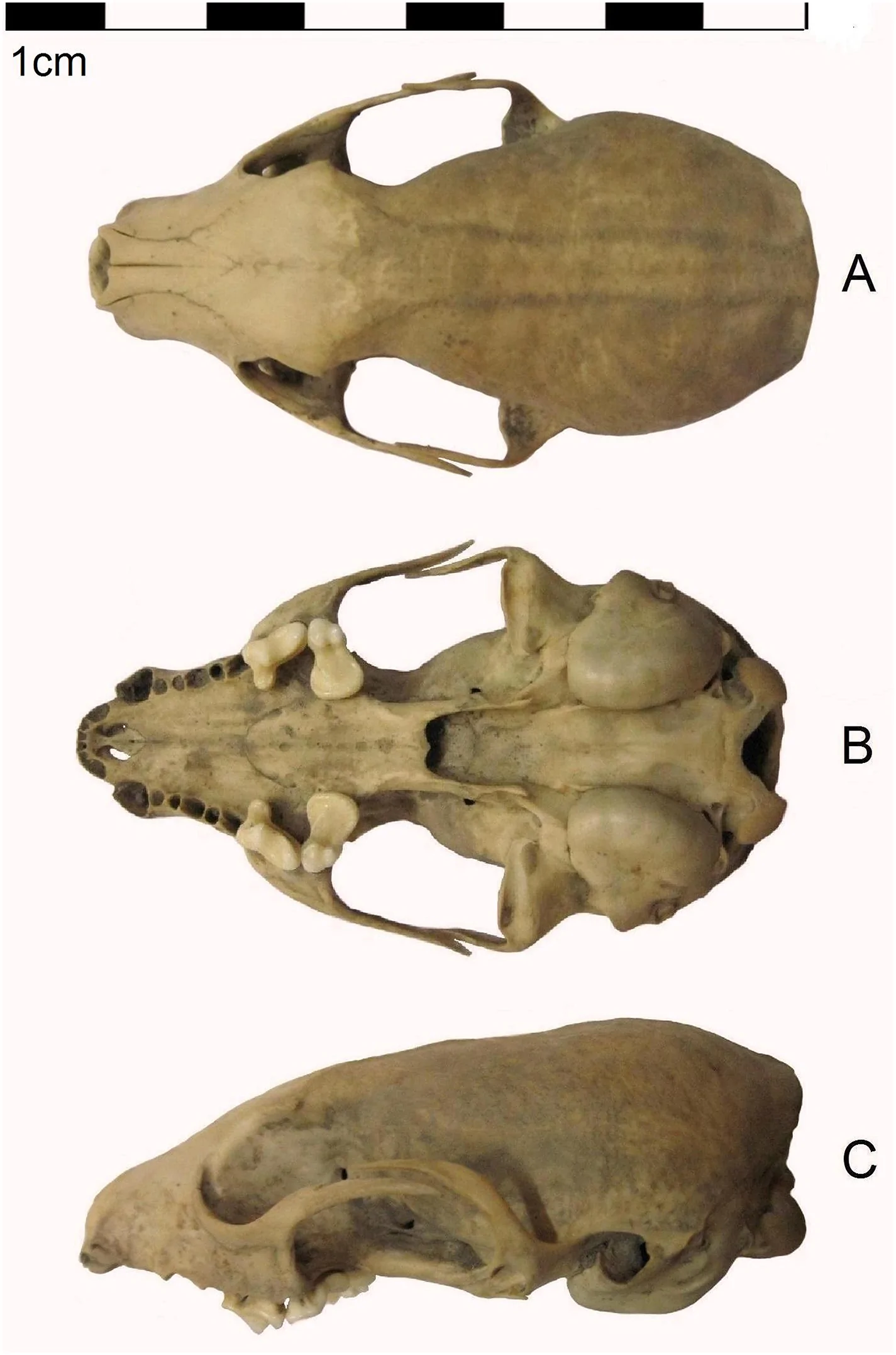
Ogorokha *Martes zibellina* cranium. dorsal (A), ventral (B) and lateral (C) views.

The color of the facial bones of the cranium is almost the same as the modern bones—milky yellowish; the brain part of the skull is somewhat darker—yellowish-light brown (Fig 5). The occipital crest on the skull is not fully developed. The temporal lines are separated and run almost parallel to each other, but in the parietal region they tend to converge. The rudiments of the sagittal ridge are weakly expressed, the occipital crest is formed. A certain dental attrition is evident. The relatively robust skull belonged to a young/sub-adult male individual aged 1–1.5 years, corresponding to the transient maturity group I/II of modern sable. A slightly higher biological age assessment (2 years and 8–10 months) suggests the degree of development of the sagittal crest—adult group III [41]. The cranial sutures are partly obliterated. The nasal bones are not fused; there is no visible superficial damage.

#### Dentalia.

For the upper jaw (Fig 5B–5C), only the left and right P^4^ and M^1^ are preserved; the remaining maxilla teeth are lost and only their alveoli are visible. The wear of the teeth is average. The morphology of the teeth does not differ from modern ones, the incisor rows are arranged in an arc, tightly adjacent to each other. Diastemas are observed between the extreme incisors and canines. The canine tooth was massive, judging by the alveolus size; the P^2^ have two roots, the premolars are straight and fit tightly together (Fig 5B).The length of the premolar P^4^ is 7.7 mm, the width of the anterior part is 5.0 mm. In the males of modern sables *M. zibellina jenisseensis* the size of the corresponding teeth is 7.7–9 mm and 4.6–5.4 mm, respectively (n = 15) (Table 1). The carnassial tooth (P^4^) does not protrude from the occlusion row; it is relatively short and massive, wide, with a good, separate and distinct protocone, the anterior edge of which is located slightly behind the anterior edge of the paracone. A distinct ridge rises to the apex of the paracone in the middle of the anterior part of the tooth crown. A distinct basal collar is traced on the lingual side of the P^4^ crown, which is most developed under the metacone. P^4^ and M^1^ are tightly pressed against each other, without diastemas. M^1^ is relatively wide without constriction in the crown, elongated in the transverse direction; the length is much less than the width. The paracone is noticeably larger than the metacone; there is a collar at the anterior outer edge of the paracone, which forms a distinct protrusion in the middle of the crown of the tooth. The paraconid and metaconid together are smaller than the protoconid (Fig 7).

**Table 1:** Skull dimensions of *Martes zibellina* (the Ogorokha Site) (Fig 4).

Measurements, mm		Fossil	Modern aboriginal population *M. zibellina jenisseensis*	
			Oleneksky district, Yakutia (Boeskorov, 2013)	
Ogorokha River, Yakutia	males, n = 15 limit X + m	females, n = 15 limit X + m
Total length		81.2	80-86.8 84.37 ± 0.44	78-85 79.02 ± 0.57
Condylobasal length		79.8	78-86.4 83.33 ± 0.53	74.5-82 77.59 ± 0.55
Basic length		74.2	74-80.4 77.74 ± 0.45	70.3-77.2 72.42 ± 0.48
Interorbital width		18.6	17.3-21.4 19.31 ± 0.30	16.8-19.4 17.91 ± 0.25
Postorbital width		17.1	14-17.1	13.8-17.6
			16.00 ± 0.29	15.23 ± 0.17
Width of canines		16.3	15.1-18 16.37 ± 0.20	14.3-16.6 15.23 ± 0.17
Length C-M1 alv.		29.1	28.7-31.4 29.96 ± 0.24	27.3-29.4 28.16 ± 0.21
LengthP1-P4 alv.		19.8	18.7-23 20.42 ± 0.29	18.2-19.7 18.95 ± 0.14
LengthP1-M1 alv.		23.2	23-26.5 24.66 ± 0.26	22-24.5 23.26 ± 0.21
P4	length	7.7	7.7-9 8.22 ± 0.08	7.2-8.4 7.67 ± 0.11
	length lingual.	8.4	8.1-9.2 8.73 ± 0.09	7.6-8.9 8.22 ± 0.12
	width anterior	5.0	4.6-5.4 5.07 ± 0.05	4.3-5.3 4.72 ± 0.09
	width posterior	2.9	2.9-3.3 3.11 ± 0.03	2.7-3.2 2.91 ± 0.04
Height at bullae timpani		30.9	29.6-32.6 30.86 ± 0.22	28.4-31.4 29.62 ± 0.23
Height in M1 region		24.8	19.1-24 21.60 ± 0.30	18.4-20.2 19.22 ± 0.13

### Taxonomy

**Fig 6:**
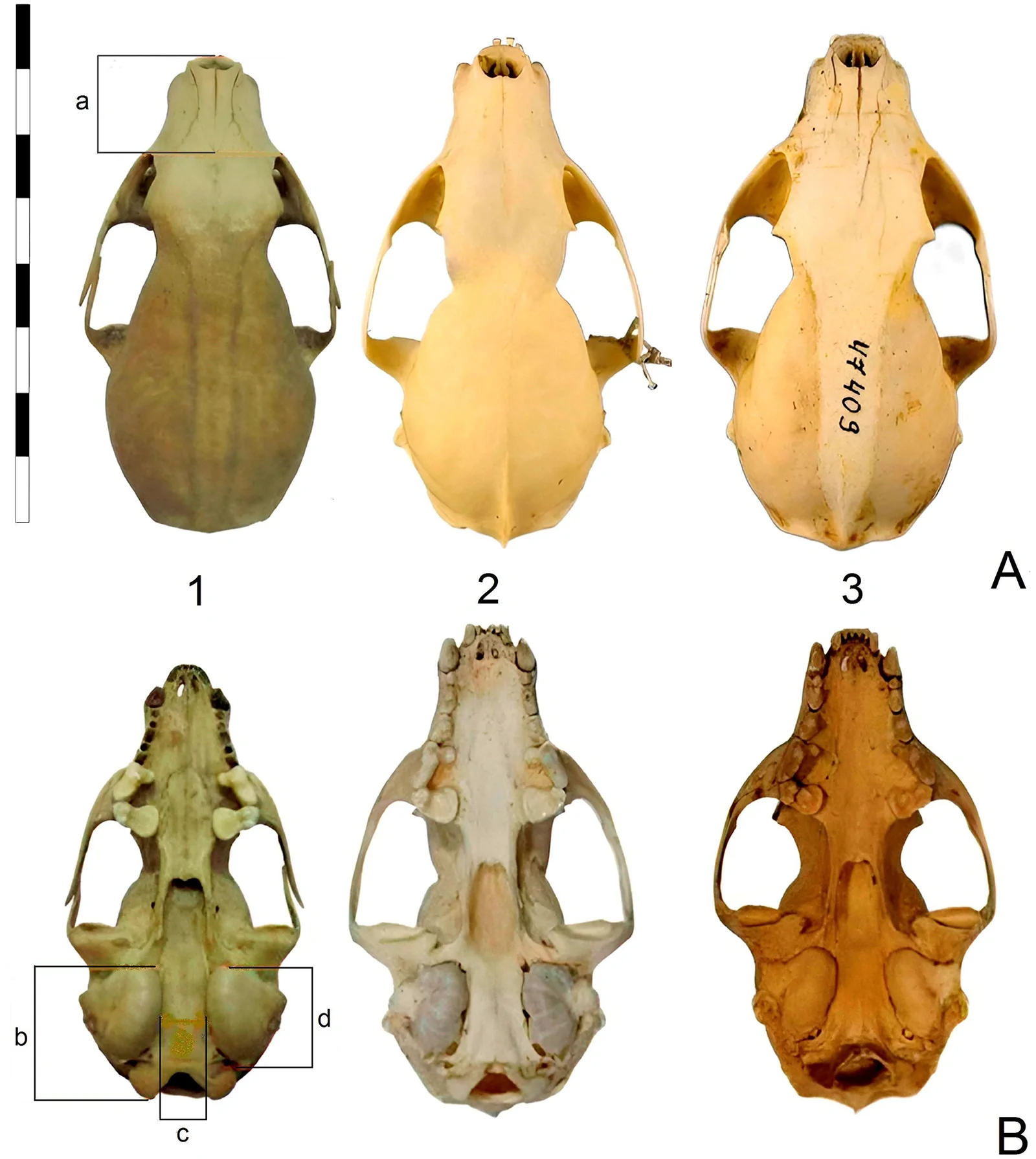
Comparison of the studied sable and marten skulls (A–dorsal view; B–ventral view). 1 – skull of the Ogorokha fossil sable, 2 – skull of a pine marten Martes martes (Linnaeus, 1758), 3 – skull of a stone marten M. foina (Erxleben, 1777) a – facial length*, b* – distance from the anterior edge of the auditory bulla to the end of the occipital condyle, *c* – distance between the posterior carotid openings, *d* – distance from the anterior end of the auditory bulla to the posterior edge of the paroccipital process.(http://szmn.eco.nsc.ru/picts/Mammalia/Martes_foina.htm). (https://skullbase.info/skulls/mammals/european_pine_marten_male/european_pine_marten_-male3.jpg) (https://skullbase.info/skulls/mammals/beech_marten/beech_marten3.jpg).

Family Mustelidae, Fisher, 1817—Mustelids Genus *Martes*, Pinel, 1792; *Martes zibellina* (Linnaeus, 1758). The morphological comparison of the fossil sable from Ogorokha with the skulls of modern martens—pine marten *Martes martes* (Linnaeus, 1758) and stone marten *Martes foina* (Erxleben, 1777) reveal marked differences (Fig 6A–6B). The Ogorokha find has characteristic features of modern sables, which distinguish them from martens [11].

**Fig 7:**
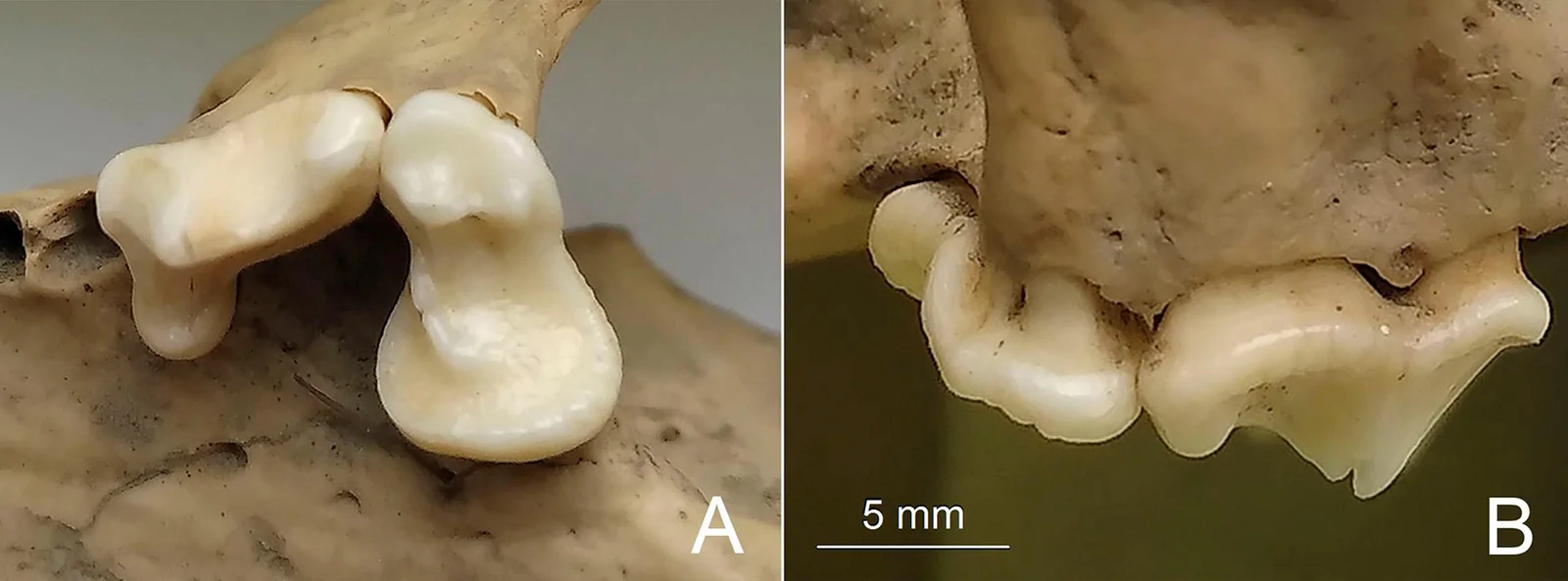
Ogorokha *Martes zibellina.* A detailed view of the preserved dentalia (P^4^ and M^1^).

**Fig 8:**
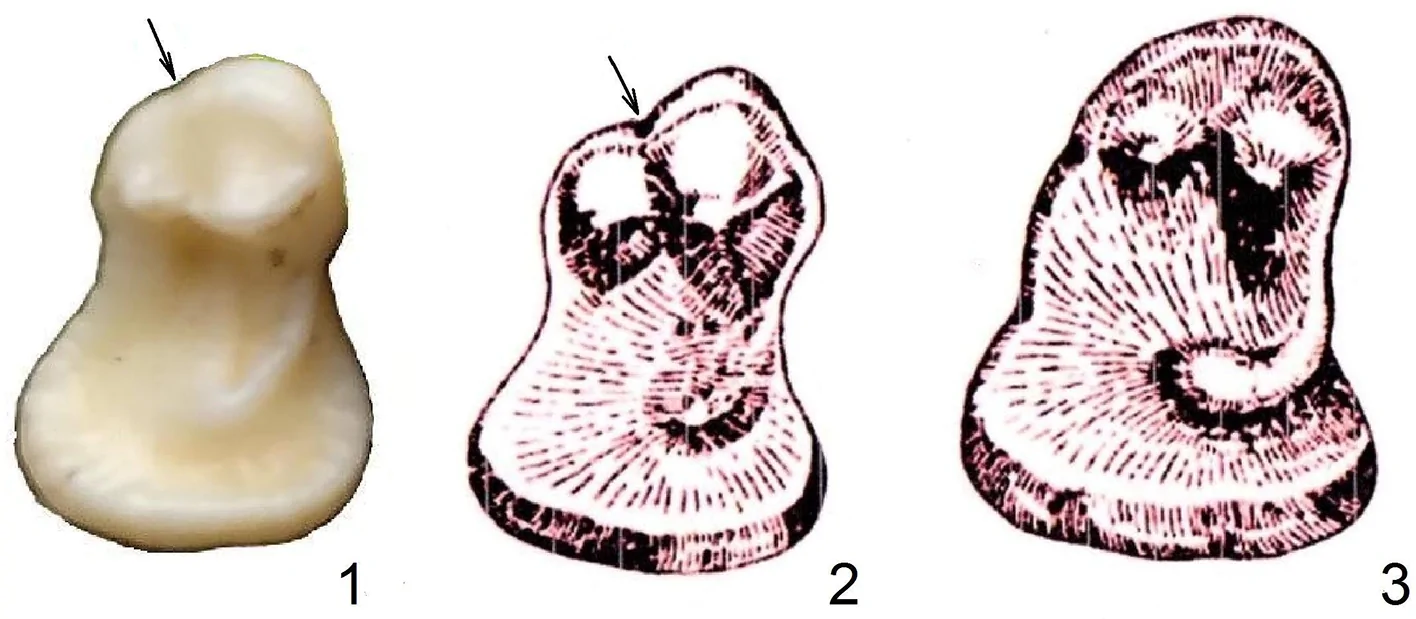
The structure of the chewing surface of the first upper molar (M1) of the fossil sable from Ogorokha (1), stone marten *Martes foina* (Erxleben, 1777) (2) and pine marten *Martes martes* (Linnaeus, 1758) (3). The arrow indicates a deepening of the vertical groove on M1. Figs 8−2 and 8-3 [11].

The facial length of the fossil skull (21.6 mm) (Fig 6A-1-a) is smaller than the distance from the anterior edge of the auditory bulla to the end of the occipital condyle (24.2 mm) (Fig 6B-1-b) At martens, these lengths’ ratio notably differ; the facial length is greater than the distance from the anterior edge of the auditory bulla to the end of the occipital condyle. The distance between the posterior carotid openings (Fig 6B-1-c) at the Ogorokha sable (9.3 mm) is less than half the distance from the anterior end of the auditory bulla to the posterior edge of the paroccipital process (10.1 mm) (Fig 6B-1-d) Martens exhibit a different lengths’ ratio: the distance between the posterior carotid openings is more than half the length of the auditory bulla [11]. On the labial side of the last molar (M1) of the Ogorokha sable, there is a clearly visible vertical groove, which brings the sable closer to the stone marten and distinguishes both species from the pine marten (Fig 7, Fig 8).

The Late Pleistocene skull of the Ogorokha sable is somewhat smaller in size comparing the skull of the modern boreal sable from the northwestern Yakutia, which belong to the relatively large subspecies *M. zibellina jenisseensis* [1,11,42] (Table 1). With respect to the assessed sub-adult age of the Pleistocene find, the Ogorokha skull along with the overall skeletal structure would likely have reached a larger size in the adulthood. On the labial side of M^1^ there is a clearly visible vertical groove, which brings the sable closer to the stone marten *Martes foina* (Erxleben, 1777) and distinguishes both species from the European pine marten *Martes martes* (Linnaeus, 1758). Based on the above morphological characteristics, the find is very close to the modern subspecies *M. zibellina jenisseensis* [43].

### Taphonomy

The skull does not show any signs of trauma; the loss of teeth occurred after the burial of the carcass. Upon discovery, the cranium showed only very minor signs of decay and aerobic superficial osteological alteration which can be explained by the millennia long permafrost burial. The present cryolithic bedrock temperature within the broader area of NE Yakutia at a depth of 15–30 m below surface varies between –3ºC and –70ºC [28]. The temperature at the ground level during the Last Ice Age was certainly much lower comparing the present. Progressive diagenesis changes in the fossil bone porosity and the chemical composition as a result of past microbial degradation and organic decay were absent [44].

Absence of a mechanical damage and a surficial attrition of the bone surface indicate just a minor relocation of the ancient sable cadaver prior to the soft-tissue decomposition of the animal in situ. Poorly oxygenated and waterlogged past geo-contextual conditions are indicated by yellowish coloring on the surface of the cranium. The overall physical state of the find shows that the sable became sealed in the Ice Age permafrost shortly after its death, and was preserved for more than 45,000 years in the hard-frozen state in the Late Pleistocene cryolithic deposits prior to its recent exposure. This conclusion corroborates the supreme physical state of conservation of other vertebrae fossils from the Ogorokha locality and the other locations within the central Indigirka area [30,45–47]. There are no signs of human-inflicted trauma or anthropological manipulation. Sable was subject of active hunting during postglacial and mainly the historical times.

## Discussion

The Ogorokha sable belongs to the group of the Paleoarctic taiga mammals [48], inhabiting dark coniferous taiga [49]. The fossil remains of the sable in Siberia are known from the Upper Pleistocene caves in the Northern and Central Urals, the Altai, the foothills of the Eastern Sayan Mountains, and the Angara River valley (Pre-Baikal region) [11]; in the Russian Far East from Primory’e [17]. The origin of sable (or its ancestor) is seen in the east of Eurasia, with spread to the northern and western parts of North Eurasia [50–52]. The genetic diversity of the mitochondrial DNA suggests a species dispersed from the Far East and later the Urals last glacial refugia [53]. Its eastern origin in the Amur River basin is supported by the morphological features such as the fur color and phenetic features of the skull [54] in consistency with the results of genetic molecular studies of the sable population structure [55]. Just sable as the only representatives of the genus *Martes* currently inhabits the present Yakutia. No fossil remains of martens have been found here.

From an evolutionary perspective, the *Martes* genus experienced a territorially framed adaptive development during the Quaternary [56]. The most ancient (~100,000 years-old) remains of sable to day in Siberia have been recorded in the Altai Mountains region [57]. The demise of the Ice Age climates promoted the northern expansion of forests into the former tundra and periglacial steppes. *M. zibellina* left refugia and spread into new territories [50,58,59]. The Late Pleistocene sable is sporadically recorded the Ural Mountains during the MIS 3 interstadial (58,000–25,000 years ago) [60–64], reaching a maximum distribution during the early postglacial times (10,000–8,000 years ago) [59]. The previous isolated finds in Yakutia date to the late Last Glacial / MIS 2 [1,18,21].

The Ogorokha sable is presently the most ancient and the northernmost located fossil Pleistocene find of this species. The skull morphology being similar of the modern sables of northwest Yakutia (the Yenisei subspecies *M. zibellina jenisseensis*) (Table 1) suggests an evolutionary lineage formed by several generations migrating from the southern parts of Siberia. The modern sables inhabiting the south of Eastern Siberia (the Eastern Sayans, the Transbaikal region, the southern Yakutia, the Amur Region and Sikhote-Alin) are relatively small in size and are often grouped into one subspecies—the Barguzin sable *(M. z. princeps)* [11]. The condylobasal length of the skull of the sable from Ogorokha is larger than the average value for adult sable males from the southern Yakutia (average 79.24 mm males, 73.31 mm females) and is close to that of the Barguzin sable (average 80.42 mm males, 78.04 mm females) [65].

The solely skull from the Indigirka site cannot yield a dimensional variability of the Late Pleistocene sable because of the deficiency of a comparative fossil material. The sable most likely reached a larger body size and had a more massive and shorter P^4^ compared with the modern species living in the same area today. The Holocene sable in the Indigirka area represents a mixture of several sub-species populations of *Martes* sp. The *M. zibellina jenisseensis* occupies today just the western part of Yakutia. There are no apparent significant differences with the modern *M. zibellina* likely because the Indigirka Ice Age animal has not reached maturity, retaining less expressed physical morphological attributes of a sub-adult individual. Studies of the present populations of *M. zibellina* in northwestern Yakutia show larger body size and a lighter fur color of the native sables comparing to the Vitim subspecies (*M. z. princeps* Birula, 1922) inhabiting the SW part of the territory (the Olekma population) [66]. The average lifespan of the wild modern Eurasian sable is ten years [67].

The discovered fossil sable from the middle reaches of the Indigirka River beyond the Polar Circle raises the question of the habitat diversity of the single *Martes* species within the specific areas of North-East Yakutia during the Last Ice Age. The high ^14^C age of the specimen suggests that sable migrated northward from the southern regions of Siberia during the warmest (early) stage of the MIS 3 interstadial within the forest zone. The forest animal likely expanded through the major (Lena, Yana, Indigirka and Kolyma) riverine systems into Arctic around 50,000 years ago [68]. Boreal woodlands—mountain foothill taiga and lowland riparian forests—along with parkland steppes formed the principal ecosystems that hosted the most productive habitats of Northeast (sub-) arctic Siberia. The extraordinarily rich and taxonomically diverse fossils of large and small fauna, including birds, document very favorable interstadial environments at the Ogorokha locality at 58,000–40,000 years ago [30]. The natural conditions might have been biotically even richer and the MAAT possibly higher than in the area today, particularly during the MIS 3 optimum (~44,500 years ago). The ancient faunal communities included some living mammal species, which are now absent in the broad geographical area, such as beaver (*Castor* sp.) apart from sable. Its presence at Ogorokha is evidenced by a beaver hut found deeply buried in the Pleistocene permafrost ^14^C-dated to *>*45,000 BP (A. Klimovskiy et al., unpublished).

Moderately cold climates with a progressing cooling trend characterized the later stage of the MIS 3 (35,000–25,000 years ago) [30,69–71]. Patchy larch *(Larix sibirica)* forests with admixture of spruce *(Picea sibirica)*, alder and birch became locally preserved. These biotopes may have provided some niches to sable prior to the onset of the late Last Glacial ~25,000 years ago. Arid tundra-steppes and open periglacial grasslands with herbs became widespread North Siberia [72]. The non-arboreal ecosystems constituted major environmental constraints to the woodland mammal. The mean annual air temperature / MAAT in East Siberia dropped by up to –10ºC during the Last Glacial Maximum / LGM (25,000–19,000 years ago), with summer temperatures by ~8–10°C lower than today and significantly lower winter temperature [73]. These adverse periglacial conditions predestined a sable retreat to more protected southern geographical locations of Siberia. Extremely cold winters reconstructed from biotic and ground-ice isotope data of North and East Siberia [74–76] may have been balanced by moderate summers due to presumed southern atmospheric flows [77]. Surprisingly, lacustrine multi-proxies from the Lake Baikal area indicate summer temperature least 3.5 °C higher than at present between 22,600–22,000 cal BP [78]. It may be speculated that the marked and regionally specific Last Ice Age climate variations may have allowed persistence of sable in some marginal forest biotopes within open periglacial landscapes [79]. The *Martes* species very likely survived in relief-protected tundra-forest and mixed taiga refugia along the northern foothills of the Sayan and Altay Mountains persisting throughout the late Last Glacial (25,000–12,000 years ago) [80,81].

The sable probably did not penetrate the eastern periglacial territories of the Western Beringia with opened arctic and sub-arctic shrub and tundra biotopes unsuitable for natural habitation of the *Martes* animals. Judging from the rare osteological finds (i.e., Dyuktay and Khaiyrgas Caves) (Fig. 1B), small populations of sable could have taken refuge in the southernmost Yakutia. More favorable maritime conditions during the LGM characterized by increased summer temperatures seem to exist in the Russian Far East—the Primory’e Region and Kamchatka, with the MAAT comparable to the modern ones or just slightly lower [82,83]. In view to the present evidence, the Late Pleistocene geographic distribution of sable was determined by climate-dependent spatial arboreal vegetation pattern strongly influenced by humidity changes rather than by seasonal temperature. The early Holocene warming pulses, leading to establishment of a compact tree cover in the former periglacial tundra lands, promoted a renewed adaptive radiation spread of *Martes zibellina* into its former northern habitats.

The main diet of this medium-size sylvan carnivore consisted of small rodents, birds and likely also fish and small amphibians as documented by modern analogues from Primory’e. Various plants, seeds and berries with their fossil residues found in the MIS 3 permafrost deposits [30,38,84] provided an additional nutrient-rich dietary resource during arctic summers. The moderate Middle and Late Pleistocene climates likely played a principal role in the phylogenetic differentiation of the sable mitochondrial gene pool. The Ogorokha sable confirms the northern spread of sable in North Asia prior to the LGM [85]. Further work, including mtDNA studies of new sable records retrieved from permafrost, will reveal more details on the geographic distribution, the time dispersal and the adaptation strategy of *Martes zibellina* within the Late Pleistocene Sub-Arctic and Arctic.

## Conclusion

The reported palaeontological find of *Martes zibellina* L. is the first one discovered in the Last Glacial Arctic. This fossil record completes the very sporadic Ice Age occurrences of this Mustelidae species from the Urals to the Russian Far East. The well-preserved specimen from the Central Indigirka broadens the documented taxonomic variability of the non-analogue small mammal community of Pleistocene Beringia. The appearance of this typical forest animal in North Siberia indicates favorable and more humid climates promoting biotically rich boreal ecosystems in the Yana-Kolyma Lowlands during the early MIS 3 interstadial. The southern retreat of taiga forests in linkage with progressing cooling towards the Last Glacial Maximum were the principal natural constraints for sable survival as of the typical woodland animal. The Ogorokha sable along with other fauna species retrieved from the Pleistocene permafrost grounds provide evidence of mosaic and biotically rich forest and parkland habitats established north of the Polar Circle ~58,000–40,000 years ago.
